# Intra- and Inter-Tumor Heterogeneity of *BRAF^V600E^*Mutations in Primary and Metastatic Melanoma

**DOI:** 10.1371/journal.pone.0029336

**Published:** 2012-01-03

**Authors:** Molly Yancovitz, Adam Litterman, Joanne Yoon, Elise Ng, Richard L. Shapiro, Russell S. Berman, Anna C. Pavlick, Farbod Darvishian, Paul Christos, Madhu Mazumdar, Iman Osman, David Polsky

**Affiliations:** 1 Department of Dermatology, New York University Langone Medical Center, New York, New York, United States of America; 2 Department of Surgery, New York University Langone Medical Center, New York, New York, United States of America; 3 Department of Medicine, New York University Langone Medical Center, New York, New York, United States of America; 4 Department of Pathology, New York University Langone Medical Center, New York, New York, United States of America; 5 Department of Public Health, Weill Medical College of Cornell University, New York, New York, United States of America; University Hospital Hamburg-Eppendorf, Germany

## Abstract

The rationale for using small molecule inhibitors of oncogenic proteins as cancer therapies depends, at least in part, on the assumption that metastatic tumors are primarily clonal with respect to mutant oncogene. With the emergence of *BRAF^V600E^* as a therapeutic target, we investigated intra- and inter-tumor heterogeneity in melanoma using detection of the *BRAF^V600E^* mutation as a marker of clonality. *BRAF* mutant-specific PCR (MS-PCR) and conventional sequencing were performed on 112 tumors from 73 patients, including patients with matched primary and metastatic specimens (n = 18). Nineteen patients had tissues available from multiple metastatic sites. Mutations were detected in 36/112 (32%) melanomas using conventional sequencing, and 85/112 (76%) using MS-PCR. The better sensitivity of the MS-PCR to detect the mutant *BRAF^V600E^* allele was not due to the presence of contaminating normal tissue, suggesting that the tumor was comprised of subclones of differing *BRAF* genotypes. To determine if tumor subclones were present in individual primary melanomas, we performed laser microdissection and mutation detection via sequencing and *BRAF^V600E^*-specific SNaPshot analysis in 9 cases. Six of these cases demonstrated differing proportions of *BRAF^V600E^*and *BRAF^wild-type^* cells in distinct microdissected regions within individual tumors. Additional analyses of multiple metastatic samples from individual patients using the highly sensitive MS-PCR without microdissection revealed that 5/19 (26%) patients had metastases that were discordant for the *BRAF^V600E^* mutation. In conclusion, we used highly sensitive *BRAF* mutation detection methods and observed substantial evidence for heterogeneity of the *BRAF^V600E^* mutation within individual melanoma tumor specimens, and among multiple specimens from individual patients. Given the varied clinical responses of patients to BRAF inhibitor therapy, these data suggest that additional studies to determine possible associations between clinical outcomes and intra- and inter-tumor heterogeneity could prove fruitful.

## Introduction

The progression of human cancers is classically thought to develop from a single mutated cell, followed by malignant clonal expansion secondary to additional genetic and genomic alterations. The continued acquisition of these alterations can result in the emergence of tumor subclones with varying phenotypic advantages (e.g. invasion, proliferation, ability to colonize different organs, etc.) [1]. Intra-tumor heterogeneity, the presence of more than one clone of cancer cells within a given tumor mass, and inter-tumor heterogeneity, the presence of different genetic alterations in different metastatic tumors from a single patient, have been identified in several tumor types [2], [3], [4], [5]. With the advent of therapies targeting specific oncogenes, it is possible to use mutation-detection strategies aimed at these oncogenes to assess tumor specimens for inter- and intra-tumor heterogeneity. Such heterogeneity is potentially important, as it has been shown to affect responses to molecularly targeted treatments in cancers such as gastrointestinal stromal tumors (GIST) and lung cancer [3], [5].

In melanoma, mutations in the *BRAF* oncogene are among the most commonly reported molecular alterations [6], [7], and BRAF is currently an exciting therapeutic target. The *BRAF^V600E^* mutation accounts for >90% of *BRAF* mutations found in melanoma [8], and confers constitutive kinase activity. Knockdown of mutant V600E expression in cultured human melanoma cell lines inhibits cell growth and invasion and promotes apoptosis [8], [9], [10], [11]. Clinical trials of selective BRAF inhibitors have shown dramatic results among melanoma patients whose tumors possess *BRAF^V600E^* mutation, but not those without the mutation, highlighting the potential clinical importance of genotyping patients' tumors to select the appropriate treatment [12], [13], [14], [15]. Most recently, the *BRAF^V600E^* inhibitor vemurafenib was shown in a phase 3 randomized clinical trial to improve overall and progression-free survival compared to dacarbazine in previously untreated patients with melanomas harboring the V600E mutation; however, a substantial majority of patients experience a partial response and progress by 8 months into treatment [12].

With the emergence of targeted therapies for melanoma it may be important to determine the extent of intra- and inter-tumor heterogeneity among primary and metastatic tumor specimens to further understand the pathogenesis of this disease and optimize treatment modalities. In the current study, we analyzed a large number of primary and metastatic melanoma tumor specimens for BRAF intra- and inter-tumor heterogeneity using a combination of 3 different BRAF mutation-detection assays as well as laser-capture microdissection. We found evidence for both intra- and inter-tumor heterogeneity of BRAF mutations within and among multiple tumors from individual patients.

## Results

### Patients and Tumors

One hundred and twelve melanoma tumors were analyzed. The study cohort consisted of 73 patients with metastatic melanoma who contributed a total of 94 metastatic tumors and 18 primary tumor specimens for analysis. Of the 73 patients, 46 (63%) were Stage III and 27 (37%) were Stage IV. Tumor specimens included 42 regional lymph node metastases, 27 regional skin metastases, 18 visceral metastases, 3 local recurrences, 3 distant skin metastases, 1 distant lymph node metastasis, and 18 primary tumors.

### BRAF mutation detection

To determine the presence of the *BRAF^V600E^*mutation in the112 melanoma specimens we utilized two techniques: conventional DNA sequencing and MS-PCR. Overall, MS-PCR detected the mutation in a greater proportion of cases than routine sequencing (**Table 1**).Using conventional sequencing we detected the mutation in 36/112 (32.1%) cases, including 7/18 (38.9%) primary tumors and 29/94 (30.9%) metastatic tumors. Mutation analysis of the same melanoma specimens using MS-PCR revealed mutations in 85/112(75.9%) cases, including 12/18 (66.7%) primary tumors and 73/94 (77.7%) metastatic tumors, indicating poor agreement between the two techniques (Kappa 0.23 for comparison of sequencing and MS-PCR in detection of mutations among metastatic specimens). Of note, there were no tumor samples in which the mutation was detected only by sequencing and not by MS-PCR. All mutations detected were V600E. Among metastatic melanomas, analysis of MS-PCR mutation status by tumor site revealed that 75% of local and regional metastases (local recurrence, regional skin and lymph node metastases) were mutant for *BRAF*, and 86.4% of distant metastases (distant skin, lymph node, or visceral metastases) were mutant for *BRAF* (p = 0.26).

**Table 1:** Detection of *BRAF^V600E^* mutations in primary and metastatic melanomas.

Melanoma	Samples (No.)	Mutations by Sequencing	Mutations by MS-PCR
Primary	18	7 (38.9%)	12 (66.7%)
Metastatic	94	29 (30.9%)	73 (77.7%)
Total	112	36 (32.1%)	85 (75.9%)

We previously demonstrated that the MS-PCR assay had a greater sensitivity to detect the *BRAF^V600E^* mutation than sequencing [16], so one explanation for the discordance in mutation rates between these techniques is that the presence of contaminating normal tissue contributed to the decreased sensitivity of mutation detection using conventional sequencing. We investigated this possibility by estimating the tumor content of each metastatic melanoma sample using light microscopy. This estimation was performed without knowledge of the mutational status of individual tumors. Cases were divided into 3 categories: <33% tumor (n = 26), 33–67% tumor (n = 19), or >67% tumor (n = 49). Using MS-PCR as the gold-standard for detecting mutations, sequencing had a sensitivity of 33% in specimens with < 3% tumor, 29% in specimens with 33–67% tumor, and 45% in specimens with >67% tumor (**Table 2**). Overall, the sensitivity of sequencing for detecting the *BRAF^V600E^* mutation was 39%., which was somewhat greater in specimens with at least 67% tumor cells.

**Table 2:** Detection of *BRAF^V600E^* mutations in melanomas grouped by tumor content.

Tumor Content	Sequencing (%)	MS-PCR (%)
<33%	8/31 (26%)	24/31 (77%)
>33%–67%	5/22 (23%)	17/22 (77%)
>67%	23/67 (34%)	51/67 (76%)
Total	36/120 (30%)	92/120 (77%)

### Laser capture microdissection to analyze intra-tumor heterogeneity

**Figure 1:**
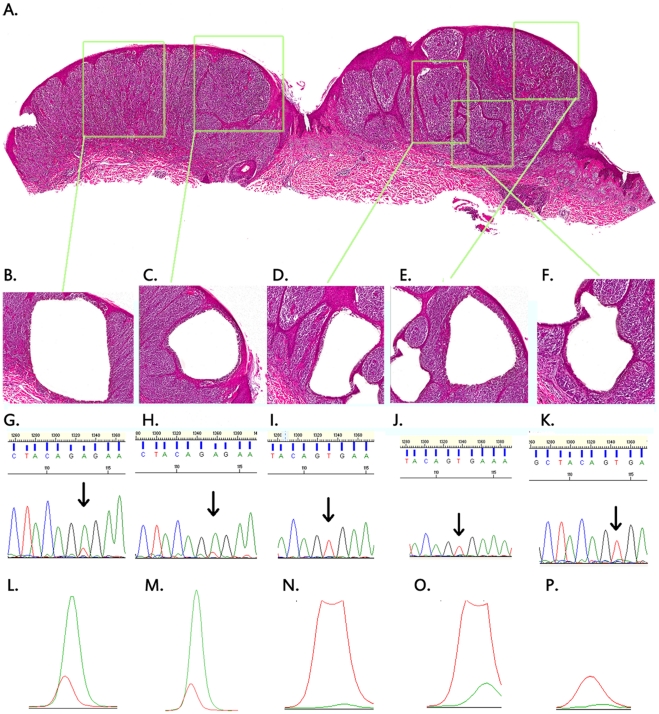
Representative Laser microdissection of a primary melanoma demonstrating intratumor heterogeneity of BRAF mutations. (**A**) Hematoxylin and Eosin stained primary melanoma with 5 areas to be microdissected marked by squares; (**B–F**) Higher magnification of microdissected areas demonstrating removal of tumor cells; (**G–K**) Sequencing electropherograms of the region of BRAF exon 15 in which the T1799A mutation is found. Each sequence is derived from the microdissected tumor region immediately above it. Note the predominant green peak of the mutant allele (A) in panels G & H, but the absence of the mutant allele in panels I, J and K; (**L–P**) Electropheogram peaks from the BRAF SNaPshot analysis corresponding to the same microdissected tumor areas as the sequencing electropherograms directly above. Note the prominent green mutant peaks in panels L & M, and the smaller but detectable mutant peaks in panels N, O and P. SNaPshot peak heights were used to estimate percentages of mutant alleles in each microdissected sample. In this example the mutant percentages were L = 81%, M = 78%, N = 5%, O = 19% and P = 14%. See Methods for additional details.

Since the discordant results from these mutation detection methods could not be accounted for by the presence of normal tissue, we speculated that individual tumors may be heterogeneous with respect to the *BRAF^V600E^* mutation, that is, the tumors may be comprised of a mixture of *BRAF^V600E^* mutant and *BRAF^wild-type^* cells, and that only the more sensitive MS-PCR could detect low numbers of mutant cells in certain specimens. To test this hypothesis, we used laser capture microdissection to isolate multiple small areas of tumor cells from each of 9 primary melanoma tumor specimens. Each dissected tumor sample was analyzed for the *BRAF^V600E^* mutation via sequencing and a mutation-specific SNaPshot assay (**Figure 1**). We developed this assay to provide a relative quantification of the proportion of mutant and wild-type alleles in a sample (see Methods for additional details). As shown in Figure 1, there were samples in which the mutant peak could be detected by the SNaPshot assay but not by direct sequencing (compare sequences in panels I, J and K to their respective SNaPshot analyses in panels N, O and P). Other investigators have also demonstrated that SNaPshot technology is more sensitive than sequencing in detecting mutations in tumor specimens [17], [18].

For each of these 9 tumor specimens, we dissected 3 to 5 regions of tumor cells and used the mutation-specific SNaPshot assay to estimate the relative proportions of *BRAF^V600E^* and *BRAF^wild-type^* DNA in each dissected area. We used these proportions to calculate the overall statistical variance in *BRAF^V600E^* DNA within each tumor. We chose the variance as a simple measure of intra-tumor heterogeneity for *BRAF^V600E^* mutant cells. The relative percentages of mutant *BRAF* DNA detected in the dissected regions from these 9 tumors ranged from 0% to 81.2%. It is important to note that the dissected areas contained between 30 and 300 tumor cells, so it is unlikely that failure to detect the mutant allele in some regions was due to sectioning of tumor cell nuclei. Based on the variance (×100) values and the distribution of the data we classified the tumors into 3 categories: those in which intra-tumor heterogeneity was “unlikely”, those in which intra-tumor heterogeneity was “likely”, and those in which the intra-heterogeneity was “marked” (**Table 3**). For example, the mutant DNA percentages from the 5 dissected regions in tumor #9 ranged from 4.9% to 81.2%. The statistical variance ×100 for this tumor was 13.691; hence this tumor was assigned to the “marked” heterogeneity category. In contrast, the mutant DNA percentages from 5 dissected regions in tumor #1 ranged from 39.4% to 56.1%. The statistical variance ×100 for this tumor was 0.419, hence it was categorized in the “unlikely” heterogeneity category. Overall, 6 of 9 primary melanomas were categorized as having “marked” or “likely” heterogeneity. This variation in the detection of the *BRAF* mutation within single tumor samples supports the hypothesis that melanomas are comprised of tumor subclones that differ with respect to the mutational status of the *BRAF* gene. It also provides an explanation for the greater sensitivity of MSPCR versus conventional sequencing in detecting *BRAF* mutations in tumor-rich samples.

**Table 3:** Detection of intratumor variation in BRAF mutation rates via laser capture microdissection.

Tumor	No. regions dissected	*BRAF^V600E^* DNA percentages					Statistical variance (×100)	Presence of heterogeneity^1^
		Dissected region						
		1	2	3	4	5		
1	5	39.4%	42.8%	43.6%	48.1%	56.1%	0.419	Unlikely
2	4	7.4%	13.4%	16.3%	31.3%		1.038	Unlikely
3	3	6.7%	7.9%	29.0%			1.575	Unlikely
4	3	0.0%	16.8%	33.4%			2.787	Likely
5	4	0.0%	21.9%	32.5%	39.7%		2.991	Likely
6	4	9.7%	42.5%	52.9%	53.6%		4.247	Marked
7	4	0.0%	0.0%	22.2%	48.3%		5.286	Marked
8	4	0.0%	0.0%	0.0%	48.9%		5.969	Marked
9	5	4.9%	13.9%	18.8%	77.7%	81.2%	13.691	Marked

1 Qualitative judgment based on variance values, see text for full explanation.

### Utilization of MS-PCR to investigate inter-tumor heterogeneity in melanoma patients

Assuming that primary melanomas contain a mixture of subclones characterized by *BRAF^V600E^* or *BRAF^wild-type^* cells, we investigated whether it was the *BRAF^V600E^* mutant clones within the tumors that preferentially metastasized. Such an observation would support a model in which BRAF was a driver of the metastatic phenotype. We analyzed a set of primary and metastatic samples from the same patient (‘matched pairs’) to answer this question. Eighteen melanoma patients had both primary and metastatic tumor specimens available for analysis. We examined the concordance in mutation status, as determined by MS-PCR, among these paired specimens. We found that 10/18 (56%) patients had tumor specimens that were concordant for the *BRAF* mutation; that is, both their primary and metastatic specimens had a mutant *BRAF* allele. Eight of 18 (44%) patients had tumors that were discordant for the *BRAF* mutation. Six of these 8 patients had wild-type primary tumors, but mutant metastatic specimens, a pattern consistent with the acquisition of the *BRAF^V600E^* mutation conferring a growth/survival advantage for metastases. Unexpectedly, 2/18 (11%) patients had *BRAF^V600E^* primary specimens but *BRAF^wild-type^* metastases (**Table 4**). As the progression to metastatic disease is generally thought to develop through the acquisition of additional genetic alterations, one would expect any and all metastatic tumors arising from a *BRAF^V600E^* primary tumor to have the same *BRAF^V600E^* mutation. Identification of *BRAF^wild-type^* metastases from *BRAF^V600E^* primary tumors supports the concept that intra-tumor heterogeneity of *BRAF^V600E^* mutant cells exists within primary melanomas.

**Table 4:** BRAF mutation concordance between primary and metastatic specimens using MS-PCR.

Patient	Primary tumor	Metastatic tumor
1	Wild Type	Mutant
2	Wild Type	Mutant
3	Wild Type	Mutant
4	Wild Type	Mutant
5	Wild Type	Mutant
6	Wild Type	Mutant
7	Mutant	Mutant
8	Mutant	Mutant
9	Mutant	Mutant
10	Mutant	Mutant
11	Mutant	Mutant
12	Mutant	Mutant
13	Mutant	Mutant
14	Mutant	Mutant
15	Mutant	Mutant
16	Mutant	Mutant
17	Mutant	Wild Type
18	Mutant	Wild Type

Assuming that both *BRAF^V600E^* and *BRAF^wild-type^* cells could give rise to metastatic tumors, we used MS-PCR detection of the *BRAF^V600E^* mutation to investigate inter-tumor heterogeneity between multiple metastatic tumors from individual patients. Nineteen patients had multiple metastases available for analysis, and a total of 40 metastatic specimens were studied. In 13/19 (68%) patients, all analyzed metastases were mutant; 1/19 (5%) patients had two wild-type metastases. Surprisingly, 5/19 (26%) patients had metastases that were discordant for the *BRAF^V600E^* mutation. These patients had both mutant and wild-type metastatic tumors (**Table 5**). Taken together, these data and the analysis of the primary tumors suggest that many primary melanomas are heterogeneous with respect to the *BRAF^V600E^* mutation, and that some of the metastasizing tumor subclones do not require the presence of the *BRAF* mutation.

**Table 5:** BRAF mutation concordance between multiple metastatic specimens using MS-PCR.

Patient	Metastasis 1	Metastasis 2
19	Wild Type	Wild Type
9	Wild Type	Mutant
20	Wild Type	Mutant
21	Wild Type	Mutant
22	Wild Type	Mutant
23	Wild Type	Mutant*
2	Mutant	Mutant
6	Mutant	Mutant
14	Mutant	Mutant
15	Mutant	Mutant
24	Mutant	Mutant
25	Mutant	Mutant
26	Mutant	Mutant
27	Mutant	Mutant
28	Mutant	Mutant
29	Mutant	Mutant
30	Mutant	Mutant
31	Mutant	Mutant*
32	Mutant	Mutant

*patient had a third metastasis which was mutant by MS-PCR.

## Discussion

The recent success of BRAF^V600E^ kinase inhibitors in melanoma has been dramatic; however, enthusiasm has been tempered by the heterogeneity and relatively short duration of patients' responses. Recent results from clinical trials demonstrate remarkably high response rates of 60% to 80% in melanoma patients with advanced metastatic disease whose tumors harbor the *BRAF^V600E^* mutation. There is a subset of patients, however, whose tumors harbor the mutation yet fail to achieve any significant response to therapy. Even among patients who benefit from BRAF inhibitors, responses, albeit profound, are generally short-lived. Resistance to the inhibitor vemurafenib, for example, usually develops within 8 months [12], [13].

To improve our understanding of melanoma biology and develop effective, personalized treatment options it is important to determine the degree to which primary and metastatic tumors result from the emergence of a dominant clone of tumor cells, or are comprised of several different malignant tumor cell clones. A recent analysis of 3 breast cancer tumors using single nucleus sequencing provides a striking example of the polyclonal nature of some primary and metastatic tumors [19]. Here we report an investigation of intra- and inter-tumor heterogeneity in melanoma using the presence of a single oncogene mutation to define the presence of a clonal population of tumor cells. We analyzed the *BRAF^V600E^* mutation because it is the most frequently detected mutation in melanoma, it has been studied extensively in pre-clinical models as a driver of the malignant phenotype(reviewed in [20]), and understanding its role in melanomagenesis is highly relevant to current clinical trials with BRAF and MAPK pathway inhibitors. Using a combination of very sensitive assays for detecting *BRAF* mutations, including laser-capture microdissection, we found substantial evidence for the presence of intra-tumor heterogeneity in primary melanomas and inter-tumor heterogeneity between multiple metastatic tumors from individual patients.

Using MS-PCR we observed a substantially higher rate of *BRAF* mutation in primary and metastatic melanoma tumor specimens compared to sequencing. These mutation rates are comparable to previously reported rates in melanoma [8], [21], [22], [23], [24], [25], [26], [27]. One explanation for the greater sensitivity of MS-PCR to detect the *BRAF^V600E^* mutation is that it is an allele-specific PCR that requires very little mutant template to return a positive result [16], [28]. Our previous work demonstrated that the sensitivity of routine sequencing to detect the *BRAF^V600E^* mutation diminishes as the proportion of *BRAF^wild-type^* DNA is a sample rises, but the sensitivity of the MS-PCR is unaffected [16]. Given these assay characteristics we expected the detection rates of sequencing to approach those of the MS-PCR in tumor-rich samples. Surprisingly, there was a marked discordance in the detection rates between the two methods even in these tumor-rich samples. This finding led us to speculate that the difference in the detection rates between DNA sequencing and MS-PCR was not due solely to the presence of contaminating normal tissue, but perhaps due to the presence of multiple tumor subclones within individual tumor specimens, some of which were *BRAF^wild-type^* while others were *BRAF^mutant^*.

We directly tested this hypothesis of intra-tumor heterogeneity using laser-capture microdissection combined with SNaPshot technology allowing semi-quantitative assessment of *BRAF^V600E^* and *BRAF^wild-type^* alleles. We found that a substantial proportion of individual tumor specimens contained a mixture of *BRAF* mutant and wild-type melanoma cells. This finding is consistent with recent data analyzing acquired melanocytic nevi, benign neoplasms of the melanocytic lineage that frequently possess the *BRAF^V600E^* mutation. Using single-cell PCR analysis, Lin et al. demonstrated that *BRAF^V600E^* mutations could be detected in different cells within the same nevus [29]. Our results are also in agreement with their more recent findings that melanomas also display intratumor heterogeneity with respect to *BRAF^V600E^* mutations. They performed single-cell PCR and sequencing of 40–56 cells from each of five primary melanomas and found that 4 of 5 primary melanomas contained both *BRAF^wild-type^* and *BRAF^V600E^* tumor cells. They supported this finding with an analysis of 10 additional melanomas using a more sensitive mutation detection assay and subcloning [30].

Although *BRAF* mutation is not required for the formation of all nevi and melanomas, it is a commonly held assumption that when present, *BRAF* mutations are a very early mutational event that cooperates with additional alterations in growth control genes to drive melanomagenesis. Together, the findings in the current study and the published data suggest a more complex picture characterized by both intra-tumor heterogeneity of primary melanomas and inter-tumor heterogeneity among metastatic tumors. Many primary melanomas appear to be comprised of at least 2 malignant subclones that differ with respect to their *BRAF* genotype (mutant or wild-type). Thus, mutations in *BRAF* do not appear to be an initiating event for all cells in a given nevus or melanoma, even those neoplasms in which the mutation can be detected, as it may be present in only a subset of tumor cells. In addition, our analysis of a set of matched primary and metastatic tumors from individual patients further suggests that *BRAF* mutations may not be required for development of metastasis in *BRAF^V600E^*-mutant primary melanomas. We observed that two primary melanoma tumors containing *BRAF^V600E^* mutations gave rise to metastases that were *BRAF^wild-type^*. Additionally, in 5 patients with multiple metastases, separate metastases from the same patient were found to be discordant with respect to *BRAF* mutation status using the highly sensitive MS-PCR assay. Based on these findings, it appears that primary melanomas may contain a heterogeneous mixture of *BRAF^V600E^* and *BRAF^wild-type^* tumor cells, and it is possible for both populations to give rise to metastases. This model is supported by an interesting report from Sensi et al in which the investigators were able to isolate, via single cell cloning, separate populations of BRAFV600E/NRASwild-type and BRAFwild-type/NRASQ61R melanoma cells from a single metastatic tumor [31].

Intratumor heterogeneity has been recognized as a general characteristic of many cancers. Importantly, it is becoming apparent that the efficacy of, and resistance to molecularly targeted therapies may be dependent upon the presence of genetically distinct tumor subclones. For example, a study of intratumor heterogeneity of *EGFR* mutations in non-small-cell lung cancer found that tumors that contain both mutation-positive and mutation-negative tumor cells are less responsive to gefitinib than tumors that do not display such heterogeneity [5]. To date, studies of BRAF resistance in melanoma have identified several mechanisms that bypass the pharmacologic block of mutant BRAF. Two of these mechanisms: 1) upregulation of COT a member of the Ser/Thr MAP3K kinase family that functions downstream of BRAF; and 2) mutation of NRAS, result in activation of the MAPK proliferation pathway [32], [33]. The *NRAS* mutation findings [33] are particularly interesting from the perspective of tumor heterogeneity. First, the two different *NRAS* mutations identified by the group were found in two distinct BRAF-resistant post-treatment nodal metastases from the same patient. This finding supports the concept that separate metastases within a single patient may be driven by different subpopulations with distinct molecular alterations, as our study suggests. Secondly, examination of the sequencing electropherogram for the sample with the mutated *NRAS^Q61K^* allele (their supplemental figure 14) reveals that the *NRAS* mutant allele appeared to be present in a very small population of cells, based on the heights of the mutant versus wild-type peaks and the investigators inability to detect the mutant peak in 3/6 macrodissected regions of the tumor specimen. In comparison, sequencing of *BRAF* in short-term cultures from the same tumor revealed an easily detected mutant peak, suggesting that more than half of the tumor was comprised of *BRAF* mutant cells. This suggests that a large proportion of *BRAF* mutant cells in the resistant tumor did not harbor the *NRAS* mutation.

Since the mutant form of NRAS can bypass BRAF inhibition [34], [35], [36], [37], [38], [39], it is surprising that such a small percentage of *BRAF^mutant^* cells carrying the *NRAS* mutation would be sufficient for conferring treatment resistance. One explanation may be that multiple mechanisms of secondary resistance allow for the concomitant survival of the *BRAF^mutant^* cells that were wild-type for *NRAS* (e.g. activation of PDGFR [33]). Indeed, there is published evidence that heterogeneity can evolve both within and between tumors from the selective pressures of molecularly targeted therapies. An analysis of separate metastatic gastrointestinal stromal tumors from individual patients to identify drug-resistance mechanisms revealed different secondary mutations in different tumors, and even found multiple secondary mutations within the same metastasis [3]. In addition, a recent study of resistance in ovarian carcinoma to platinum-based chemotherapy has supported this model of multiple, intrinsically resistant subclones present at initial tumor presentation, prior to treatment [40].

As mechanisms of resistance to BRAF inhibitor therapy continue to be uncovered, it will be prudent to consider the implications of intratumor heterogeneity on treatment response and management. The potential presence of heterogeneity among metastatic tumors suggest that relying on a single biopsy specimen for treatment decisions with BRAF inhibitors could exclude some patients who would benefit from the therapy. These patients essentially have a “false negative” biopsy result, as different, non-biopsied metastatic tumor sites may harbor a *BRAF* mutation [41], [42]. Genotyping a second tumor specimen from a patient whose initial results are negative for the *V600E* mutation may reduce the possibility for such “false negative” genotyping results. An alternative genotyping approach being explored by our group is the use a blood-based mutation-detection method that would assay DNA shed from all metastatic sites, not just a single tumor. This approach could potentially expand the pool of patients eligible for these drugs.

Another implication of the results described here is the possibility that very small populations of *NRAS^mutant^/BRAF^wild-type^* tumor cells may co-exist with *BRAF^mutant^* tumor cells within the same patient. This possibility is further supported by recent data from a large retrospective study of *BRAF* and *NRAS* genotyping results demonstrating a small fraction of patients in which both *BRAF* and *NRAS* mutations were detected in their tumor specimen, possibly due to intratumor heterogeneity [43]. The co-existence of *BRAF^mutant^* and *NRAS^mutant^* tumor cells has potential clinical implications as in-vitro studies have demonstrated that pharmacologic inhibition of wild-type BRAF in the presence of oncogenic RAS can promote melanoma proliferation and/or resistance to apoptosis [34], [35], [36], [37], [38], [39]. Presumably the treatment of patients with a mixed population of tumor cells could result in the rapid development of treatment resistance as has been observed in some patients. As activation of the MAPK pathway appears to be one of the critical elements contributing to melanoma cell proliferation and survival [38], [44] current strategies to combat resistance to BRAF inhibitor therapy are focused on combination therapies that simultaneously target more than one component of the MAPK pathway, such as BRAF and MEK (NCT01072175). Given the potential activating effects of BRAF inhibition in the presence of oncogenic NRAS, and the potential for mutations in downstream pathway members such as MEK1 [44], it may become important to assess genetic heterogeneity among tumor specimens both before treatment and upon disease recurrence to more fully understand the development of resistance to targeted therapies.

In conclusion, data from the current study and the published literature support a model in which individual melanoma tumors can be polyclonal, that is, comprised of a mixture of cells that may or may not have the *BRAF^V600E^* mutation, with both populations having the ability to metastasize. These findings warrant further study in the context of melanoma tumor progression models, and whether the degree of intratumor heterogeneity in patient tumors could influence the efficacy of molecularly targeted therapies directed against mutant BRAF. The growing body of evidence demonstrating intratumor heterogeneity within solid tumors, including melanoma, suggests that selecting the optimal therapeutic regimen for melanoma patients may ultimately hinge on characterizing tumors based on the precise genetic makeup of tumor subclones.

## Materials and Methods

### Ethics Statement

The study was approved by the New York University School of Medicine Institutional Review Board, and all patients signed written informed consent at time of enrollment.

### Patients and Tumor characteristics

Melanoma patients were prospectively accrued in the New York University School of Medicine Interdisciplinary Melanoma Cooperative Group database. Patients had either stage III or IV disease at time of enrollment. Patients were selected based on the availability of clinical specimens, and all available tumor tissue from each patient was included in this study. Laser capture microdissection and *BRAF* T1799A SNaPshot analysis was performed on 9 primary melanoma tumors that were selected out of convenience, that is, tumor blocks were readily available for the tissue sectioning required for laser capture microdissection.

### Mutation detection using whole tissue sections

Unstained cut sections mounted on slides were scraped into 1.5-ml microcentrifuge tubes. Subsequent steps followed the protocol for DNA isolation from paraffin slides in the QIAmp Mini Blood DNA kit (Qiagen, Valencia, CA). For each melanoma tumor, hematoxylin and eosin-stained slides were reviewed to ensure enough viable tumor, and each slide was scored for tumor content by two investigators (DP and MY) to estimate the percent of tumor nuclei to all nuclei in the sample. Scores were defined as <33% tumor, 33%–67% tumor, and greater than 67% tumor. The investigators were blinded to the mutation results at the time of assessment of tumor content. For conventional sequencing, amplification of the entire *BRAF* exon 15 was accomplished as previously described, using primers designed by Davies, et al. [8], [22]. Mutant-specific polymerase chain reaction (MS-PCR) was performed as previously described [16]. In addition, DNA from the human melanoma cell line SK-MEL 29 (mutant for *BRAF*) was used as a positive control, and human placental DNA was the negative control.

For the MS-PCR all tissue specimens were run in duplicate. In the event of discordant results, mutation status was determined by a third run. There were 20 cases in which there was discordance between the initial 2 MS-PCR reactions. Of these, 10 cases resulted in a positive mutation call on the subsequent run, and 10 cases resulted in a wild-type call. Each run was accompanied by a dilutional series of control DNA ranging from 0.001 ng SK-MEL 29 to 10 ng SK-MEL 29 in triplicate as a quality control measure of assay sensitivity. Human genomic placentalDNA (Sigma-Aldrich, St. Louis, MO), wild type for *BRAF*, and water were included in every run in triplicate as negative controls.

### Laser capture microdissection

Freshly cut 10 micron paraffin-embedded sections from nine primary melanomas were placed onto polyethylene naphthalate (PEN) membranes that were mounted onto glass slides (Leica, Wetzlar, Germany). Sections were stained with hematoxylin and eosin (H&E) for 30 minutes to allow for visualization of nuclei and cytoplasm and maintained without cover slips as required for laser capture microdissection (LCM). Cover-slipped H&E slides previously cut from the same block of tissue were examined using light microscopy to identify tumor rich regions lacking invading lymphocytes or interspersed fibrous stroma which could contain contaminating normal fibroblasts. Laser-assisted microdissection of melanoma cells was performed using the Leica Microsystems LMD7000 laser capture microdissection system. The smallest dissected area measured approximately 1500 µm^2^, the largest approximately 15,000 µm^2^, corresponding to approximately 30 to 300 cells in each dissected section. A minimum of 3 and a maximum of 5 dissected areas were obtained from each case; in 5/9 (56%) cases 4 areas were dissected. DNA was extracted and purified from each dissected area separately using the Qiagen QIAmp DNA micro kit according to the manufacturer's instructions. Purified DNA was used as a template to amplify *BRAF* exon 15 using the following primers: 5′- AGTAACTCAGCAGCATCTCAGG and 5′-ATCTCTTACCTAAACTCTTCATAATGC. This set of primers creates a 273 bp amplicon, which was used for sequencing and mutation detection using SNaPshot technology.

#### Use of SNaPshot technology to detect BRAF T1799A hot-spot mutation

Amplified *BRAF* PCR products were purified using PCR Clean-up Kits (Roche Molecular Biochemicals, Indianapolis, IN) and subjected to SNaPshot reaction according to the manufacturer's instructions (Applied Biosystems, Foster City, CA) using the following probe: 5′-CACAGTAAAAATAGGTGATTTTGGTCTAGCTACAG. The products of the SNaPshot reaction were examined via gel-capillary electrophoresis using an Applied Biosystems ABI310 genetic analyzer and data was interpreted using Genemapper software (Applied Biosystems). The percentage of mutant allele was calculated by the following formula:
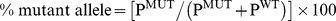
where P^MUT^ is the peak height of the mutant allele and P^WT^ is the peak height of the wild-type allele.

Based on the distribution of the data, we grouped the cases into 3 categories. The cases with the 3 lowest variances were grouped into a category in which substantial tumor heterogeneity was unlikely (variances = 0.419, 1.038, and 1.575, respectively). The case with the next highest variance (Case #4) had a value that was nearly double the value of specimen with the highest variance in the “unlikely” category. Therefore, we grouped Case #4 and those with higher variance values into the category where substantial tumor heterogeneity was likely or even marked.

### Statistical Analysis

Descriptive statistics were calculated for demographic and clinicopathologic characteristics. The concordance (agreement) between direct sequencing (wild type/mutant) and MS-PCR (wild type/mutant) was assessed by the kappa statistic. The sensitivity and specificity of direct sequencing, utilizing MS-PCR as the gold standard, were estimated to examine the false positive and false negative rates of direct sequencing. The assessment of agreement between direct sequencing and MS-PCR was also stratified by tumor content (<33%, 33–67%, and >67%). The chi-square test was used to assess the relationship between tumor site (local/regional vs. distant) and MS-PCR mutation status (wild type/mutant). All p-values are two-sided with statistical significance evaluated at the 0.05 alpha level. Ninety-five percent confidence intervals (95% CI) were calculated to assess the precision of the obtained estimates. All analyses were performed in SAS Version 9.1 (SAS Institute Inc., Cary, North Carolina) and Stata Version 10.0 (StataCorp, College Station, Texas).
